# A retrospective study to validate an intraoperative robotic classification system for assessing the accuracy of kirschner wire (K-wire) placements with postoperative computed tomography classification system for assessing the accuracy of pedicle screw placements

**DOI:** 10.1097/MD.0000000000004834

**Published:** 2016-09-23

**Authors:** Tai-Hsin Tsai, Dong-Syuan Wu, Yu-Feng Su, Chieh-Hsin Wu, Chih-Lung Lin

**Affiliations:** a Graduate Institute of Medicine, College of Medicine, Kaohsiung Medical University; b Department of Neurosurgery, Kaohsiung Medical University Hospital; c Department of Neurosurgery, Kaohsiung Municipal Hsiao-Kang Hospital; d Graduate Institute of Clinical Medicine, College of Medicine, Kaohsiung Medical University, Kaohsiung, Taiwan.

**Keywords:** accuracy, bone-mounted miniature robotic system, pedicle screw

## Abstract

This purpose of this retrospective study is validation of an intraoperative robotic grading classification system for assessing the accuracy of Kirschner-wire (K-wire) placements with the postoperative computed tomography (CT)-base classification system for assessing the accuracy of pedicle screw placements.

We conducted a retrospective review of prospectively collected data from 35 consecutive patients who underwent 176 robotic assisted pedicle screws instrumentation at Kaohsiung Medical University Hospital from September 2014 to November 2015. During the operation, we used a robotic grading classification system for verifying the intraoperative accuracy of K-wire placements. Three months after surgery, we used the common CT-base classification system to assess the postoperative accuracy of pedicle screw placements. The distributions of accuracy between the intraoperative robot-assisted and various postoperative CT-based classification systems were compared using kappa statistics of agreement.

The intraoperative accuracies of K-wire placements before and after repositioning were classified as excellent (131/176, 74.4% and 133/176, 75.6%, respectively), satisfactory (36/176, 20.5% and 41/176, 23.3%, respectively), and malpositioned (9/176, 5.1% and 2/176, 1.1%, respectively)

In postoperative CT-base classification systems were evaluated. No screw placements were evaluated as unacceptable under any of these systems. Kappa statistics revealed no significant differences between the proposed system and the aforementioned classification systems (*P* <0.001).

Our results revealed no significant differences between the intraoperative robotic grading system and various postoperative CT-based grading systems. The robotic grading classification system is a feasible method for evaluating the accuracy of K-wire placements. Using the intraoperative robot grading system to classify the accuracy of K-wire placements enables predicting the postoperative accuracy of pedicle screw placements.

## 1 Introduction

Posterior instrumentation, such as pedicle screws,^[1]^ transfacet screws,^[2]^ or translaminar facet screws,^[3]^ is commonly used to supplement anterior lumbar interbody cage fusion.^[4]^ Transpedicular screws are steadier than other spinal fixation techniques.^[4]^ Pedicle screw implantation can be verified through intraoperative fluoroscopy and postoperative plain radiography or computed tomography (CT).^[5]^ Although CT scans are the golden standard for evaluating the accuracy of pedicle screw placements,^[5]^ accurate positioning in most patients is verified through intraoperative fluoroscopy and postoperative plain radiography, which are less accurate^[6,7]^ and rely on the surgeon's judgment of whether screw placements are clinically acceptable.

The Renaissance robotic system (Renaissance, Mazor Robotics Ltd, Caesarea Park South, Israel) guides surgeons in implanting pedicle screws. Although the Renaissance robotic system has been used to assist with pedicle screw placements for more than 10 years, surgeons have not used it as a diagnostic tool. Kuo et al^[8]^ first introduced a classification system through secondary registration to evaluate the intraoperative accuracy of Kirschner wire (K-wire) placements and classified the screw placements to improve the accuracy. However, the rationale and effectiveness of intraoperative robotic grading system for predicting the postoperative accuracy of pedicle screw placement is unknown. This purpose of this retrospective study is validation of an intraoperative robotic grading classification system for assessing the accuracy of K-wire placements with the various postoperative CT-base classification systems for assessing the accuracy of pedicle screw placements.

## 2 Materials and methods

### 2.1 Study sources

**Figure 1:**
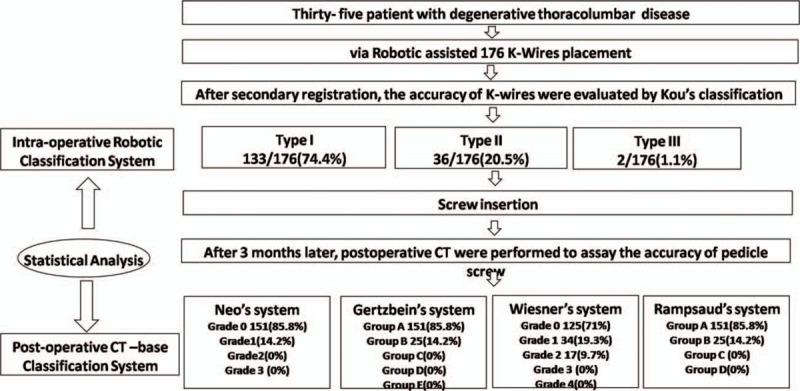
Flowchart of the study design.

We conducted a retrospective review of prospectively collected data from 35 consecutive patients who underwent 176 robotic assisted pedicle screws instrumentation at Kaohsiung Medical University Hospital from September 2014 to November 2015. The flowchart of study procedure is shown in Fig. 1.

### 2.2 Selection of participants

Surgical indications include failure of conservative treatment, ongoing neurological deficit, intractable back pain, and progressive deformity. Inclusion criteria consisted of patient having diagnosed of degenerative thoracolumbosacral disease such as degenerative disc disease, facet disease, and degenerative scoliosis; (age > 20 years at the time of diagnosis; refractory to conservative treatment for 6 months; corrected with robotic-assisted pedicle screw placement; and CT was followed at 12 months. Exclusion criteria consisted of prior trauma; infection; malignancy; and adult degenerative scoliosis.

### 2.3 Ethics statement

This clinical study was approved by the Institutional Review Board of Kaohsiung Medical University Hospital: (No: KMUHIRB-E(I)-20150167). Written informed consent was obtained from all the participants.

### 2.4 Surgical techniques

**Figure 2:**
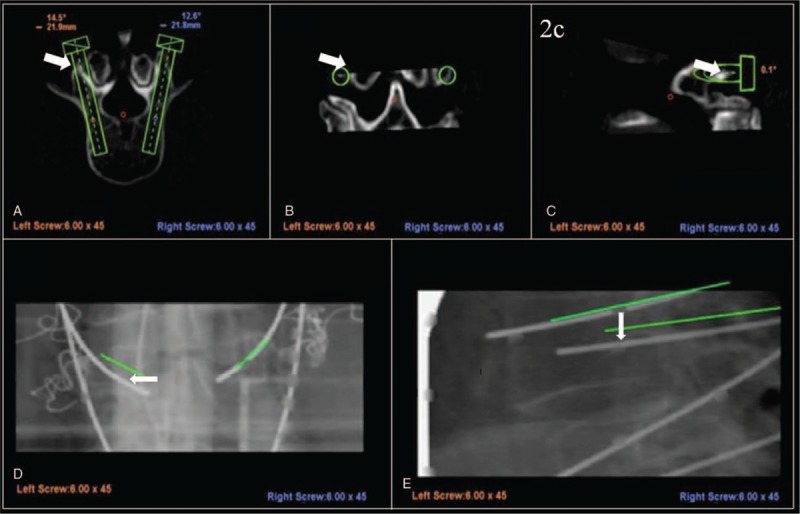
A preoperative planning entry point at the slope of the bony surface causes inferior lateral skiving during drilling execution (A–C). After the re-registration step, the left K-wire is categorized as Type III (deviation > 3 mm), and the right K-wire categorized as Type I (D, E). K-wire = Kirschner wire.

All the surgeries were performed by single surgeon. Surgical procedures of the Renaissance robotic system include preoperative planning, mounting, primary registration, robot assembly, drilling execution, K-wire insertion, secondary registration, and pedicle screw insertion (Fig. 2).

### 2.5 Surgical outcomes

**Figure 3:**
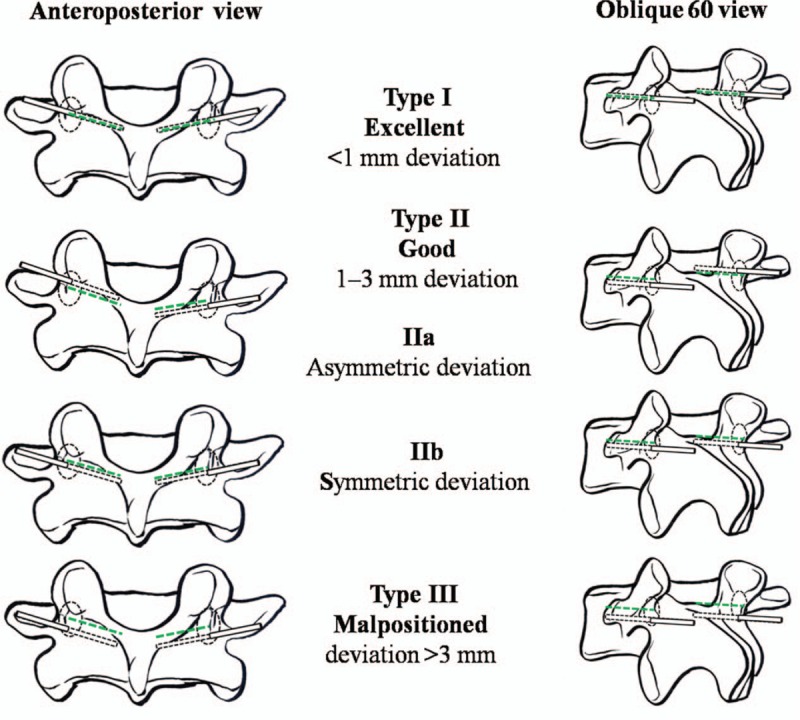
Classification system for the accuracy of pedicle screw placements with a bone-mounted miniature robotic system. The green dashed line indicates the preoperative planning tract, and the black dotted line represents the intraoperative K-wire tract. K-wire = Kirschner wire.

Intraoperative Accuracy of K-Wire Placement: K-wire placements were classified into 3 types (Fig. 3).^[8]^ The robotic grading system classified unacceptable pedicle screw placements as Type III, acceptable pedicle screw placements as Type I and II.

Postoperative CT Assessment of the Pedicle Screws: Three months after surgery, the accuracy of the pedicle screw placements was assessed through CT. Neo et al^[9]^ classified unacceptable pedicle screw placements as grade 3, acceptable pedicle screw placements as grades 1 and 2, and optimal pedicle screw placements as grade 0. Gertzbein and Robbins^[10]^ classified unacceptable pedicle screw placements as grades D and E, acceptable pedicle screw placements as grades B and C, and optimal pedicle screw placements as grade A. Wiesner et al^[11]^ classified unacceptable pedicle screw placements as grades 3 and 4, acceptable pedicle screw placements as grades 1and 2, and optimal pedicle screw placements as grade 0. Furthermore, Rampersaud et al^[12]^ classified unacceptable pedicle screw placements as grade D, acceptable pedicle screw placements as grades B, C, and optimal pedicle screw placements as grade A.

### 2.6 Statistical analysis

To assess the postoperative accuracy of pedicle screw placements, we used the common CT-base classification system such as Neo system, Gertzbein system, Wiesner system, and Rampersaud system according to the degree of deviation (Table 1). The distribution of accuracy between the intraoperative robot-assisted and various postoperative CT-based classification systems was compared using kappa statistics of agreement. The accuracy before and after repositioning and secondary registration were compared using the McNemar test. Statistical analysis was performed using SPSS version 19 for Windows (IBM, New York). All statistical results with *P* <0.05 were considered statistically significant.

**Table 1:**
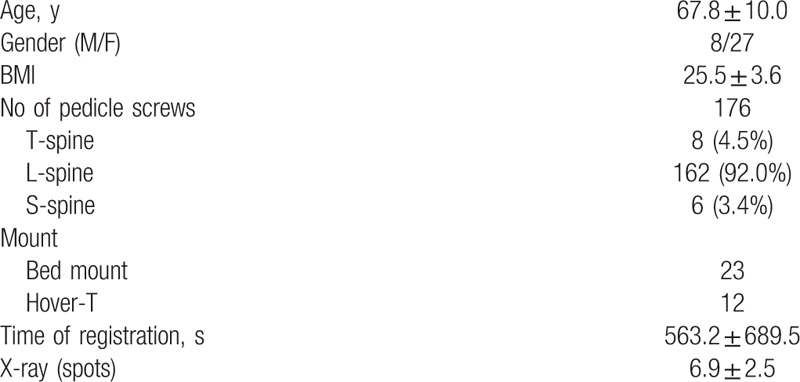
Clinical characteristics of 35 patients with robot-guided pedicle screw placement.

## 3 Results

### 3.1 Clinical characteristics

Thirty-five consecutive patients were scheduled for thoracolumbar spine surgery with posterior instrumentation for degenerative diseases, in which the Renaissance robotic system was used to place pedicle screws (Table 1). We evaluated a total of 176 pedicle screw placements in all the patients. The mean patient age was 67.8 ± 10.0 years, and 27 patients (77%) were women. Most screws were placed in L4 and L5 (Table 2).

**Table 2:**
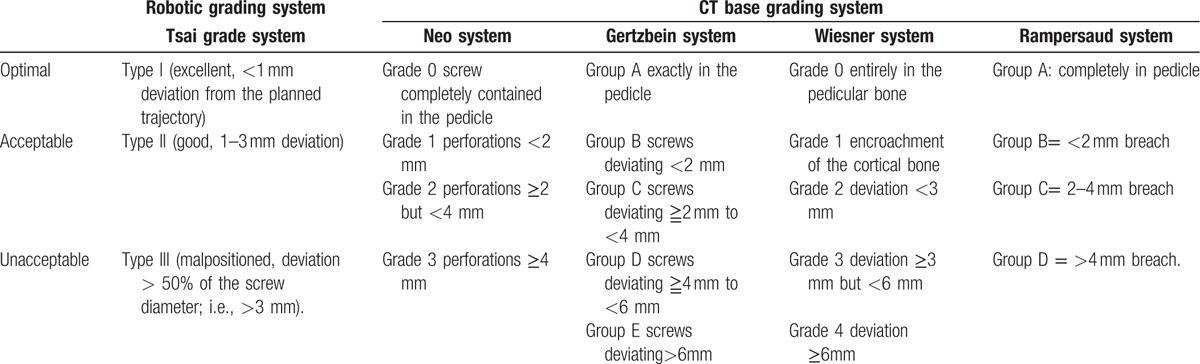
Grading system of the pedicle screw placement.

Table 3 shows the distribution of the accuracy of the pedicle screw placements according to various classification systems. In the systems proposed by Neo et al,^[9–12]^ Gertzbein and Robbins,^[9–12]^ and Rampersaud et al,^[9–12]^ 85.8% of the screw placements were evaluated as optimal and 14.2% were evaluated as acceptable. In the system proposed by Wiesner et al,^[9–12]^ 71.0% of the screw placements were evaluated as optimal and 29.0% were evaluated as acceptable. No unacceptable screw placements were observed in the aforementioned studies.

**Table 3:**
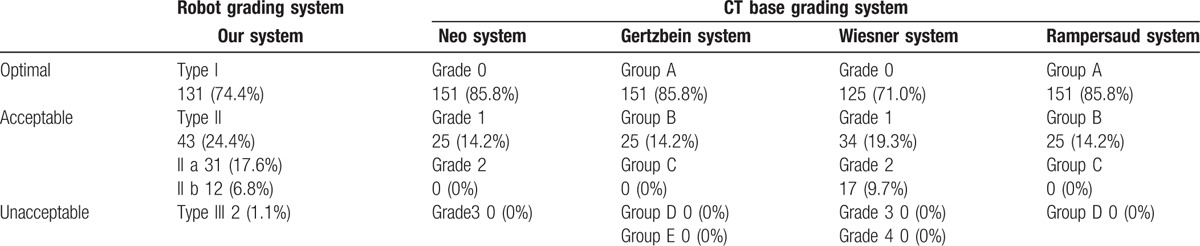
Pedicle screw placement grading systems.

The distribution of the intraoperative accuracy of K-wire placement is shown in Table 4. After secondary registration, the intraoperative K-wire position was matched to the preoperatively planned position of pedicle screws, and the accuracy of the K-wire placements was classified into 3 types. Before and after repositioning, the placements were classified as excellent (131/176, 74.4% and 133/176, 75.6%, respectively), satisfactory (36/176, 20.5% and 41/176, 23.3%, respectively), and malpositioned (9/176, 5.1% and 2/176, 1.1%, respectively). Acceptable K-wire placements accounted for 94.9% and 98.9% of all placements (inclusive of types I and II) before and after repositioning, respectively. The Mc Nemar test revealed a significant difference (*P* = 0.0082; Table 4).

**Table 4:**
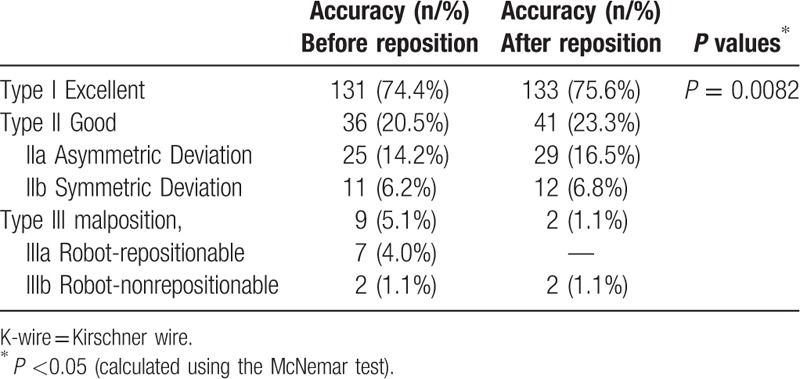
Classification of the accuracy of 176 K-wire placements through secondary registration before and after repositioning.

**Figure 4:**
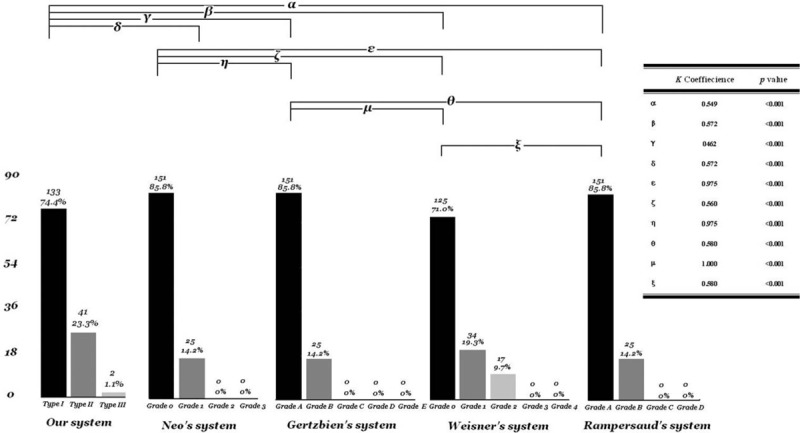
Distribution of the accuracy between robot-assisted grading classification and various CT-based classification systems. CT = computed tomography.

A comparison of the distribution of accuracy between the robotic grading system and various CT-based classification systems is shown in Fig. 4. No significant difference was observed between the robotic grading system and various CT-based classifications, namely the grading systems reported by Rampersaud et al^[12]^ (kappa coefficient of agreement, κ = 0.572, *P* <0.001), Wiesner et al^[11]^ (κ = 0.462, *P* <0.001), Gertzbein and Robbins^[10]^ (κ = 0.572, *P* <0.001), and Neo et al^[9]^ (κ = 0.549, *P* <0.001), indicating that the robotic grading system and those proposed in previous studies have significantly relevant assessment results and high consistency. These results show that the robotic grading system is useful for evaluating the intraoperative accuracy of K-wire placements, and its sensitivity is comparable to that of various CT-based classification systems.

## 4 Discussion

The Renaissance robotic system for pedicle screw implantation has been shown to demonstrate satisfactory accuracy.^[13–16]^ The use of this system for placing pedicle screws has yielded accuracies of 95% to 99% in previous studies.^[16–18]^ Ringel et al^[17]^ reported an accuracy of 95% for pedicle screws placed using this robotic system. Devito et al^[16]^ reported an accuracy of 98.3% in 635 patients from 14 medical centers. Roser et al^[18]^ reported an accuracy of 99% for pedicle screws placed using this system. For K-wire placements that deviated ≤3 mm from the planned trajectory, the accuracy was improved from 94.9% to 98.9% after repositioning through secondary registration. This shows that the Renaissance robotic system is a precise tool that can assist in pedicle screw implantation with a satisfactory level of accuracy.

We developed a robotic grading classification system to evaluate the intraoperative accuracy of K-wire placements by using the Renaissance robotic system. CT is the golden standard for evaluating the accuracy of pedicle screw placements.^[5]^ However, CT is seldom used to determine the accuracy of pedicle screw placements in clinical settings, and most patients continue to undergo radiography examinations. Examining the accuracy of pedicle screw placement through fluoroscopy or radiography requires experienced surgeons, and these methods are less accurate than CT.^[6,7]^ Despite these advantages in using CT, it has the shortcoming of exposing patients to more radiation than other methods do. By contrast, using the Renaissance robotic system to evaluate screw placements is more accurate and does not expose patients to additional radiation. Therefore, this robotic system can assist in precisely placing the pedicle screws and may serve as a tool for diagnosing pedicle screw accuracy.

The kappa coefficient of agreement was used to validate the differences between the robotic grading system for K-wire placement and CT-based grading systems for pedicle screw placement. After validation, no significant difference was observed, as described in the Results section. Kappa statistics revealed that the sensitivity of the Renaissance robotic system is comparable to that of the various CT-based grading systems. Therefore, it can be used to determine the accuracy of pedicle screw placements. The results of the kappa statistics for the association between the intraoperative robotic grading classification and postoperative CT-based classification systems are statistically significant, implying that the intraoperative robotic grading system of K-wire placement is reliable for predicting the accuracy of postoperative pedicle screw placements.

According to the strategies for malpositioned screws in this system, immediate repositioning is suggested when the K-wire deviates >3 mm (type III) from the planned trajectory. This robotic grading classification system enables the precise and objective assessment of the degree of deviation; however, it results in a high intraoperative repositioning rate. Without secondary registration, Hu et al^[19]^ immediately repositioned 1% of screws during surgery. Through secondary registration, Kuo et al^[8]^ immediately repositioned 5.99% of screws during surgery, and the rate of intraoperative misplacement of K-wires also reached 9/176 (5.1%) in that study. On the basis of these studies, the robotic grading system increased the intraoperative reposition rate. As previously reported, screw loosening is highly associated with repeated repositioning.^[20,21]^ However, in this study, malpositioned K-wires were repositioned before pedicle screw placements were performed. The pedicle screw tracts were not overly tapered or repeatedly augmented. Therefore, a high intraoperative reposition rate was less related to screw loosening, and the incidence of screw loosening under the robot grading system warrants long-term follow-up.

After validation, we considered the robotic grading system a feasible method for precisely evaluating the accuracy of K-wire placements through secondary registration. Despite this system offering several benefits to users, only a few surgeons use it. First, the system analyzes the degree of deviation during surgery. Second, it determines whether intraoperative repositioning is necessary. Third, it determines the cause of deviation and immediately adjusts the K-wire. Finally, the system predicts the postoperative accuracy of pedicle screw placements. Therefore, this system is a useful method for evaluating the accuracy of K-wires placements; it not only increases the accuracy of intraoperative K-wire placements, but also predicts the postoperative accuracy of the placement.

The differences between the robot-assisted classification for K-wires placements and various CT-based classifications for pedicle screw placements were compared under the kappa statistics of agreement. We ruled out a malpositioned screw, so the statistics could be implemented without affecting the overall statistical results. No significant difference was observed, indicating that the robotic system and those proposed in previous studies have significantly relevant assessment results and high consistency. However, CT scans are still the golden standard for evaluating the accuracy of pedicle screw placements. Although only a few surgeons use it, the classification system is an alternative method for assessing the accuracy of pedicle screw placements by using a bone-mounted miniature robotic system. Further research on the correlation of pedicle screws between the robotic system and postoperative CT is warranted.

## 5 Conclusion

Our findings revealed no significant differences between the novel robotic grading system and various CT-based grading systems. The robotic grading system is a feasible method for evaluating the accuracy of pedicle screw placements. Using the intraoperative robotic grading system to classify pedicle screw placements facilitates predicting the accuracy of the placement.

## Acknowledgment

The authors thank Professor Yi-Hsin Yang for the specialized assistance in the statistical analysis.
